# Right/left ventricular area ratio does not correlate with right ventricular impedance

**DOI:** 10.1186/cc10822

**Published:** 2012-03-20

**Authors:** F Paalvast, D Reis Miranda, M Knook, A Rossi, J Van Bommel, D Gommers

**Affiliations:** 1 Erasmus Medical Centre, Rotterdam, the Netherlands; 2 Reinier de Graaf Ziekenhuis, Delft, the Netherlands

## Introduction

The right ventricular/left ventricular (RV/LV) area ratio is often used to titrate airway pressure during mechanical ventilation [1]. This ratio has only been validated for detecting pulmonary embolism [2]. The purpose of this study was to evaluate the relationship between RV/LV area ratio and RV impedance in a pig model with a closed pericardium.

## Methods

Eight anesthetized pigs were instrumented with closed pericardium for the measurement of arterial blood pressure, central venous pressure, RV and pulmonary pressure. On the main pulmonary artery, an ultrasonic flowprobe (MA14PAX; Transonic) was positioned to obtain pulmonary flow. Distally, a balloon occluder was positioned facilitating gradual constriction of the pulmonary artery. To obtain a stepwise pressure difference increment over the banding we gradually inflated the balloon occluder. Occluder resistance is computed as the systolic right ventricle pressure minus systolic pulmonary pressure divided by cardiac output times 79.9. An ECG-gated CT scan of the heart was performed, 10 minutes after each banding. The RV/LV area ratio was computed by reconstructing the CT images to a typical echocardiographic four-chamber view and dividing the RV area by the LV area. All measurements were performed in triplicate and averaged. As repeated measurements in eight independent animals were used, a significant relation was sought between RV/LV area ratio and occluder resistance with ANOVA. Correlation was obtained by a Spearson's correlation.

## Results

Cardiac output dropped from 4.4 ± 0.8 to 1.7 ± 0.9 l/minute. The relations between occluder resistance and RV/LV area ratio during diastole and during systole were not significant (*r *= 0.48 resp. *r *= 0.54).

## Conclusion

The RV/LV area ratio does not correlate with right ventricular impedance in this closed pericardium model.
